# Neural Field Continuum Limits and the Structure–Function Partitioning of Cognitive–Emotional Brain Networks

**DOI:** 10.3390/biology12030352

**Published:** 2023-02-23

**Authors:** Kevin B. Clark

**Affiliations:** 1 Cures Within Reach, Chicago, IL 60602, USA; kbclarkphd@yahoo.com; 2 Felidae Conservation Fund, Mill Valley, CA 94941, USA; 3 Campus and Domain Champions Program, Multi-Tier Assistance, Training, and Computational Help (MATCH) Track, National Science Foundation’s Advanced Cyberinfrastructure Coordination Ecosystem: Services and Support (ACCESS), https://access-ci.org/; 4 Expert Network, Penn Center for Innovation, University of Pennsylvania, Philadelphia, PA 19104, USA; 5 Network for Life Detection (NfoLD), NASA Astrobiology Program, NASA Ames Research Center, Mountain View, CA 94035, USA; 6 Multi-Omics and Systems Biology & Artificial Intelligence and Machine Learning Analysis Working Groups, NASA GeneLab, NASA Ames Research Center, Mountain View, CA 94035, USA; 7 Frontier Development Lab, NASA Ames Research Center, Mountain View, CA 94035, USA & SETI Institute, Mountain View, CA 94043, USA; 8 Peace Innovation Institute, The Hague 2511, Netherlands & Stanford University, Palo Alto, CA 94305, USA; 9 Shared Interest Group for Natural and Artificial Intelligence (sigNAI), Max Planck Alumni Association, 14057 Berlin, Germany; 10 Biometrics and Nanotechnology Councils, Institute for Electrical and Electronics Engineers (IEEE), New York, NY 10016, USA

**Keywords:** classical and quantum computation, classical and quantum networks, functional brain connectivity, Hebbian and antiHebbian-type rules, neural field theories, preferential attachment rules, structural brain connectivity, synaptic scaling, topographic network theory

## Abstract

**Simple Summary:**

Pessoa postulates that bran anatomy associated with the processing and expression of emotion-laden content, such as the amygdala and limbic cortices, is resource capacity-limited. Thus, brains require multichannel or parallel structure-function connectivity to effectively perceive, motivate, integrate, represent, recall, and execute cognitive-emotional relationships. Pessoa employs 2D graph network theory to support his views on distributed brain organization and operation, concluding that brains evolve through dual-process competition and cooperation to form highly embedded computational architectures with little structure–function compartmentalization. Low-dimensional graph theory has become a popular mathematical tool to model, simulate, and visualize evolving complex, sometimes intractable, brain networks. Graph theory offers advantages to study and understand various biological and technological network behaviors and, for Pessoa, it permits a framework that accounts for structure–function features thus far poorly explained by perhaps “traditional” perspectives, which advocate for the mapping of structure–function relationships onto well-localized brain areas. Pessoa nonetheless fails to fully appreciate the significance of weak-to-strong structure-function correlations for brain dynamics and why those correlations, caused by differential control parameters such as Hebbian and antiHebbian neuronal plasticity, are best assessed using neural field theories. Neural fields demonstrate that embedded brain networks optimally evolve between exotic computational phases and continuum limits with the accompaniment of some network partitioning, rather than unconstrained embeddedness, when rendering healthy cognitive-emotional functionality.

**Abstract:**

In *The cognitive-emotional brain*, Pessoa overlooks continuum effects on nonlinear brain network connectivity by eschewing neural field theories and physiologically derived constructs representative of neuronal plasticity. The absence of this content, which is so very important for understanding the dynamic structure-function embedding and partitioning of brains, diminishes the rich competitive and cooperative nature of neural networks and trivializes Pessoa’s arguments, and similar arguments by other authors, on the phylogenetic and operational significance of an optimally integrated brain filled with variable-strength neural connections. Riemannian neuromanifolds, containing limit-imposing metaplastic Hebbian- and antiHebbian-type control variables, simulate scalable network behavior that is difficult to capture from the simpler graph-theoretic analysis preferred by Pessoa and other neuroscientists. Field theories suggest the partitioning and performance benefits of embedded cognitive-emotional networks that optimally evolve between exotic classical and quantum computational phases, where matrix singularities and condensations produce degenerate structure-function homogeneities unrealistic of healthy brains. Some network partitioning, as opposed to unconstrained embeddedness, is thus required for effective execution of cognitive-emotional network functions and, in our new era of neuroscience, should be considered a critical aspect of proper brain organization and operation.

## 1. Introduction

In *The cognitive-emotional brain: From interactions to integration*, Luiz Pessoa [1] tries to convey to readers the emergent higher-order performance benefits of embedded structure-function relationships that form across distributed brain loci which traditionally have been thought to specialize in cognitive and/or emotional processing. Pessoa’s conceptual framework, a contemporary derivative of Karl Spencer Lashley’s equipotentiality [2,3] and Rafael Lorente de Nó’s pleuripotency [4], follows the more-or-less trending movement in the neurosciences that seeks to replace outdated, restrictive notions of cognition, emotion, attention, memory, and other brain functions as being strictly isolable to brain regions [5,6,7]. He instead identifies the (primate) brain’s cytoarchitecture as a familiar set of discrete areal and laminar networks capable of modifiable dedicated functions which increase their combinatorial and computational complexity (see Glossary of Terminology) via short- to-long-range neural, humoral, and additional sorts of spatiotemporal interactions [8,9,10,11,12]. Irrespective of the contemporariness and general accuracy of his exposition, Pessoa offers few new insights about the principles and quantification of network behavior, and further disappoints us by failing to satisfactorily discuss limit-imposing deterministic and probabilistic structure-function primitives or control functions detailing real oft-interacting physiological phenomena, such as bidirectional synaptic (meta)plasticity [10,13,14], cell fate and migration [15,16], directed axonal/dendritic growth and pruning [17,18,19,20], and trophic, immunological, and developmental neuronal modulation [21,22,23,24]. The absence of this content, which is so very important for understanding the dynamic structure-function embedding and partitioning of brains, diminishes the rich competitive and cooperative, or author-coined “push-pull” and “working-together”, nature of neural networks, thus trivializing Pessoa’s own arguments on the phylogenetic and operational significance of an optimally powerful integrated brain filled with both strong and weak neural connections [25]. Indeed, commenting on this material, for which a large amount of support exists in the literature [5,7,26,27], can only enhance one of Pessoa’s secondary propositions advocating the improbability of one-to-one smooth mappings between brain structure and function–mappings that need to be addressed in our new era of neuroscience.

## 2. What Is the “Standard Network View” of Brain Structure and Function?

Pessoa devotes entire chapters and ancillary passages to elaborate (un)directed graph theory descriptions of scalable node and path characteristics in cognitive-emotional network topography. However, his premise that functional connectivity or embeddedness is “the influence elements have on the activity of other elements, which depends on structural embeddedness, in addition to other synaptic and cellular properties, ongoing activity, neuromodulators, and the like” [1] (p. 216) highlights a broader viewpoint left unexplored throughout his book. Perhaps these oversights originate from Pessoa’s narrow modern emphasis on topographic network theory, where the importance of structural and functional neural networks of any scale as units of brain processing is superior to local computational features of, for example, individual neurons, glia, and macromolecules. Or, perhaps they originate from his interest in clarifying controversial and believed-to-be physically indeterminate model-free correlative functional networks [28], which are interpreted as effectively connected networks that remain regularly independent of structural change and brain parenchyma presumably unassociated with task performance and motivational contexts (see [1], p. 209, 212, and 216). Nevertheless, inattention to transient-to-enduring plastic (sub)cellular contributions to brain organization and operation seemingly leads Pessoa to curious and somewhat ironic conclusions about what he calls the “standard network view” of robust cognitive-emotional brain functionality. Such functional networks emerge from weak-to-strong structural network connectivity, and Pessoa [29] (p. 27) obfuscates this data-supported conclusion with his marginally validated dismissal of the imprecise, function-obscuring experimental practices of binning. Binning is a popular method usually performed to improve analytical and numerical tractability or signal detection power within the spatiotemporal sampling-error and threshold limits of nonstationary noisy brain images and other forms of high-throughput digitized data functions [5,30]. When applied to weakly correlated or (experimenter-defined) subthreshold resting-state neural activity, binning may yield binary or fuzzy structure-function classification intervals that are less sensitive to functional event detection [31]. This drawback, however, represents technical constraints on executing practical, precise quantification, and is not one of methodology conceptualization, leaving suprathreshold resting-state activity also susceptible to mischaracterization depending on implemented binning parameters.

Pessoa [29] (p. 27) further criticizes the “standard network view” in the *Précis of The cognitive-emotional brain* as being one in which “network states depend on strong structural connections; conversely, weak connections have a relatively minor impact on brain states,” assertions which he later rightly acknowledges conflict with fairly recent experimental findings [32] demonstrating that “*weak pairwise correlations* are capable of generating *strongly correlated network states*”. Though Pessoa agrees with Mantini et al. [33] and Tyszka et al. [34] that massive polysynaptic projections and bioamine modulatory systems can account for cognitive-emotional functional connectivity, he inadequately appreciates these and other forms of intra- and internode communication (e.g., astrocyte support networks, trophic biochemical systems, genetic/epigenetic/somatic regulatory networks, etc.), which may dramatically elevate or reduce the degree and, accordingly, the strength of structure-function embeddedness through numerous (convergent and divergent) neural and aneural pathways (see [1]) (p. 177, 184, and 204). For Pessoa to also insist that weaker anatomical connections and labile subthreshold (resting or intrinsically connected) network-state correlations, for instance, are routinely assumed by a quorum of neuroscientists to exert little-to-no influence on network (structure-function) status is misleading and contrary to the data-driven postulates of experience-dependent plasticity championed by theorists and empiricists alike for nearly seven decades [35,36,37,38,39,40,41]. Credited as being an early, if not the first, pioneer of local plasticity rules for synapses and neurons, Donald Hebb [38] (p. 66), relying on Lorente de Nó’s previous insights, contemplated the idea that pairwise weakly correlated (subthreshold, threshold, suprathreshold, or combinations of each) activity at local neurons (i.e., transient “activity trace”) may prime proportionally stronger functional macroscopic cell assembly correlations in shared neighboring and distant fields. Many similar experimentally authenticated claims, such as that regarding synaptic scaling, by both noted and largely forgotten scientists, further challenge Pessoa’s notions about what is the true accepted “standard” perspective of brain network behavior and the role of structural change in structure-function embeddedness [28,42,43,44].

## 3. Scalability of Local Bidirectional Metaplasticity in Neural Fields

At particular issue, given Hebb’s (albeit older and incomplete) rationale and Pessoa’s denunciation of the so-called “standard network view” stressing strong connections over weak ones, is what Pessoa [1] (p. 209) refers to as effectively connected dynamic functional networks dependent on task and motivational contexts “without concomitant modification of structure.” Pessoa here self-inconsistently ignores possibilities that weak connections and structural embeddedness could actually contribute to network behavior. To illustrate his hypothetical construct, he cites, among other cases, the example of the increased coherent functional integration of potent affective information by early retinotopic human visual areas measured with haemodynamic-sensitive fMRI [45]. However, the evoked-response magnitude also intensified during functional integration, as reported by Damaraju et al. and, in contrast to Pessoa’s interpretation of unaltered gross brain anatomy, this probably caused and/or was caused by unquantified (instead of indeterminate) activity-dependent structure-function modifications at embedded sublevels of neuronal compartments (e.g., soma, dendrites, and axon) and microcircuits. Different, oftentimes interacting, local bidirectional plasticity rules account for such effects [46,47,48,49], including preferential Hebbian- and antiHebbian-type rules and additional physiologically derived axioms appropriate for the interactions and integration of scalable brain networks. For (natural and artificial) neurons obeying diverse special classes of Hebbian- and antiHebbian-type rules, mechanisms of functionally meaningful local structural change are well-known to be induced, maintained, and/or modulated across timescales of milliseconds to days by certain intensity-dependent processes associated with arousing catecholamines and peptide compounds that were likely active during testing by Damaraju et al. [45]. These kinds of local structural changes, such as those belonging to nonlinear Hebbian [41,50,51,52,53], dynamic stochastic synapse [54], spike timing-dependent plasticity [37,39,55], and quantum bidirectional plasticity learning models [10,13], are capable of mediating weak local correlative activity, which can impact global network organization and operation of, for instance, cortical sensory maps important for hierarchical, overlapping, coupled, and (semi)modular cognitive-emotional embedding [56,57,58,59].

An attractive manifestation of such scalable local bidirectional neuronal plasticity is synaptic scaling [60,61], where Hebbian-like synaptic remodeling of comparatively discrete dendritic volumes, which alone are incapable of effecting persistent overall changes in neuron discharge rates and patterns, either increases or decreases the short- to long-term responsiveness of the entire dendritic network (and, consequently, the cell and linked cell groups) through co-stimulated homeostatic protein synthesis, gene regulation, and cellular trafficking. Synaptic scaling emerges to assist in normalizing the thermodynamically-sensitive exotic (classical or quantum) statistical destabilization of competitive and cooperative compartmental interactions (e.g., winner-takes-all, fit-get-rich, first- mover-advantage, etc.) observed for integrate-and-fire neurons associated with Hebbian rules [10,13,21,60,61,62,63,64]. Through these more-or-less local processes involving both excitatory and inhibitory feedback/forward regulation, initially weakly correlated distributed dendritic and subthreshold somatoaxonal activity at one or a few neurons may begin to selectively drive stabilizing cooperative and competing structure-function variations in larger neuronal populations. Such processes, in turn, profoundly affect widespread information conduction under emotional or motivational contexts, as exemplified by reinforced spike timing [48,65,66], potentiated neuron signal tone [48,67,68], and resting membrane and action potential threshold sliding [46,58,69].

## 4. Local Parameters force Continuum Limits on the Partitioning and Embedding of Cognitive-Emotional Neural Fields

The normalizing effects of local bidirectional metaplastic events on neurons and microcircuits help to tune and constrain the performance advantages of embedded cognitive-emotional brain networks. The current state of knowledge about these structure-function continuum limits remains poor, a fact unrecognized by Pessoa [70,71,72,73,74]. Powerful neural field theories [62,75,76,77,78,79,80], equally worthwhile for data conditioning, analysis, and simulation, may be employed to interrogate the impacts of local control variables, extrapolating far more cutting-edge biological conclusions and testable predictions about cognitive-emotional networks than is possible with simpler graph-theoretic analyses of brain activity. The high-dimensional Riemannian construction of field theory models, including neuromanifolds mapped by mean field, relativistic Shannon-Einstein, and quantum field theories, has long held favor among computational neuroscientists due to its embodiment of the essential local/global organization and (functional) activity of natural neural networks [25,75,79]. Model success relies on the systematic inclusion of multiscale, physiologically relevant known and hidden neural control parameters, such as metaplastic Hebbian and antiHebbian rules or other probabilistic controls, as elements in embedded local structure-function subspaces. Distributions of weak-to-strong network connections, and therefore of performance optimization and degrees of network partitioning and embeddedness, for various computational phases progress between thermodynamic bounds set by the *energy* or *fitness* level of each node that are contained within a neuromanifold, where learning rules determine information storage capacity, processing efficiency, associativity, sparseness, modularity, and other critical structure-function specifications of random, small-world, and scale-free brain systems [5,8,10,13,81,82,83,84,85,86].

Depending on learning rate, network behavior may evolve during classical or quantum computational states [8,10,13,75,81,82,83,84,87,88,89,90,91]. Only for continuum extremes of probabilistic quantum or symmetry-associated deterministic classical behavior do synaptic or nodal clusters reach compacted densities that lead to large, sustainable, peak-degenerate structure-function map homogeneities unrepresentative of actual neural networks and healthy brains [8,13,62,63,75,76,84,91,92]. Such conditions signify deficient or absent metaplastic control, as observed with traditional two-variable nonstochastic Hebbian-type rules [54,56,93], that plague natural and artificial macroscale networks with excessive synaptic densities, insufficient information-storage capacities, and numerous pattern-retrieval errors. These and additional field theory attributes of (non)random variation in neuronal activity and hidden layers reveal the organization of structurally ambiguous functional connectivity [94,95,96] and imply that some network partitioning, as opposed to unconstrained embeddedness, is required for the maximum execution of cognitive-emotional network functions, such as fast and accurate memory searches and retrieval, learning fuzzy and discrete categories, cogency and soundness of inferences, and computational complexity of insight and analysis problem-solving [8,10,13,62,76,83,84,86,91,96,97].

## 5. Continuum Limits on a Hybrid Classical-Quantum Model of Neural Fields

Neural fields and the cognitive-emotional attributes of such fields may be derived through the statistical mechanics of natural emergent classical and quantum computation [8,10,13,84]. A set of well-known conditions, termed preferential attachment rules, emulate the Hebbian-like and nonHebbian-like synaptic or nodal plasticity rules represented in natural and artificial systems, and are thus suitable for the present purposes [8,81,82,84,98,99]. Preferential attachment rules of complex technological networks [87,88,89,90] and biological networks [81,82,98,99] obey classical Maxwell-Boltzmann, quantum Fermi-Dirac, and quantum Bose-Einstein statistics, which dictate continuum limits on the system comprised of classical and quantum neurons and synapses [100,101,102,103,104,105,106,107,108].

### 5.1. Local Control Parameters limit Structure and Function of Hybrid Classical-Quantum Neural Fields

Hebbian-like and nonHebbian-like fitness models designate a unique local fitness parameter, *η_i_*, for each network node, *i*: {*ℓ* = 1, 2, …, *i*}. The fitness parameter, *η_i_*, along with the number of existing links, *k_i_*, gained previously by node *i,* describe that node’s probability, Π*_i_*, of acquiring new links, *m*, where:

Π*_i_* = (*η_i_ k_i_*)/(Σ*_ℓ_ η_ℓ_ k_ℓ_*) (1)

Each node is also assigned a unique, fixed local energy or fitness level, *ε_i_*, defined as:

*ε_i_* = − (1/*β*)log *η_i_*, (2)

where *β* = 1/*T* and absolute temperature *T* (or population size or time corresponding to the metaphorical treatment of temperature) operates as a control variable for transitions between Bose-Einstein condensate and “fit-get-rich” computational phases. Lower *ε_i_* levels tend to collect more links. Connections between any two different nodes correspond to noninteracting, inert particles packing the energy level of each node, so that 2*m* particles become equally distributed at some time *t_i_* of the network’s evolution. The partition function *Z_t_*, relating system microstates to thermodynamic parameters, yields an occupation number,

(3) $$k_{i}\left(\varepsilon_{i},t,t_{i}\right)\;=m{\left(t/t_{i}\right)}^{ƒ(\varepsilon_{i})},$$

specifying the number of connections a node arising at time *t_i_* with energy *ε_i_* has amassed by a later time *t*. As such, the partition function *Z* behaves in a manner similar to Hebbian and antiHebbian rules, normalizing nodal energy and occupation across the composite, macrostate, or grand canonical ensemble probability distribution [109]. In standard classical Hebbian theory for nonhomosynaptic and nonhomeoplastic events within discrete or continuous training epochs,

(4) $$w_{ij}{}_{}^{}{=}_{}^{}\left(1/p\right)\sum_{p=1}^{k=1}x_{i}^{k}x_{j}^{k}$$

where weight *w_ij_* is the strength of the connection between neuron *j* and neuron *i*, *p* is the number of iterative training, and *k* is the number of neuron inputs for training set *x*. Temporal changes in *w* may be represented as correlation or covariational matrices with the rate of change driven by the typical linear combination of neuron inputs. Improved, more general forms of Equation (4) have been built to account for nonlinear and antiHebbian learning traits. These include a reformulated weighting rule stated as an activation or activity function *z*,

(5) $$\pm_{}^{}z_{i}{}_{}^{}{=}_{}^{}\sum_{i=0}^{N-1}w_{i}x_{i}+b$$

where *N* is the input neuron, *w_i_* is the connection weight for output neuron *x_i_*, and *b* is the nonlinear bias associated with neuron function. A quantum neuron, e.g., Ref. [110], also may be developed from Equations (4) and (5) with the activation function *z*:

(6) $$z_{}{}_{}^{}\equiv_{}^{}{\langle \psi_{x}|\psi_{w,b}\rangle }_{}^{}{=}_{}^{}(\vec{w}\cdot \vec{x}+b)/(N+1)$$

where classical orthogonal variables from Equation (5) are represented as quantum state vectors having modifiable probability amplitude coefficients residing in matrices or on quantum spheres [111]. For the hybrid classical-quantum model, which may involve classical and quantum neurons, the rate at which a node increases its network links proceeds via a power law established from the exponent,

(7) $$ƒ\left(\varepsilon_{i}\right)\;=e^{-\beta }{}^{\left(\varepsilon_{i}-\mu_{i}\right)},$$

where *μ* equals the chemical potential associated with free energy, and helps define energy states, gradients, and transition or switching thresholds between cognitive-emotional states, macrocircuits, microcircuits, and nodes. The power law determines continuum boundaries on the neural field which are forced through local control parameters capable of epitomizing hetero- and homosynaptic plasticity, excitatory and inhibitory plasticity, and meta- and homeoplasticity. When ƒ(*ε_i_*) = 1, Bose-Einstein statistics hold [112,113], generating the occupation number for particles or connections populating level *ε_i_*,

(8) $$n\left(\varepsilon_{i}\right)\;=\;1/(e^{\beta (\varepsilon_{i}-\mu_{i})}-1).$$

These constraints compel node *i* to maintain its vast proportion of network links. Such quantum synaptic or nodal clustering can render highly compacted densities leading to large, sustainable structure-function map homogeneities or unconstrained embeddedness unrepresentative of biological neural networks and healthy brains. Extremely strong network connections, if persistent, become pathological, preventing flexible structure-function compartmentalization and, therefore, the dynamic structure-function behavior needed for adaptive brain anatomy and processes. Neuropsychiatric conditions presenting perseveration, such as obsessive–compulsive disorder, offer biological instances of dysfunctional homogeneities involving local-to-global brain organization and operation. For ƒ(*ε_i_*) << 1, the Bose-Einstein statistical distribution switches to one described by classical Maxwell-Boltzmann statistics [112,113], resulting in the occupation number:

(9) $$n\left(\varepsilon_{i}\right)\;=\;1/e^{\beta (\varepsilon_{i}-\mu_{i})}.$$

Classical statistics characterize “fit-get-rich” and “first-mover-advantage” computational phases for classical neurons and synapses which approach zero connectivity in the thermodynamic limit [87]. Like extremely strong network connectivity, zero connectivity also might be pathological when occurring over protracted durations, perhaps contributing to overly heterogeneous structure-function relationships often linked to neuropsychiatric conditions presenting disorganized or fragmented thought patterns, such as schizophrenia. In addition, the occupation number defined by Fermi-Dirac statistics [112,113],

(10) $$n\left(\varepsilon_{i}\right)\;=\;1/(e^{\beta (\varepsilon_{i}-\mu_{i})}+\;1).$$

appears when ƒ(*ε_i_*) >> 1. Common features of Fermi-Dirac-governed networks include degenerate collections of quantum synapses and nodal groups and activity patterns capable of supporting effective multistable cognitive-emotional brain representation and operation. Importantly, although neural fields may perform better (or worse) under certain statistical conditions, hybrid classical-quantum fields promote the greatest opportunities for highly adaptive structure-function connectivity and improved cognitive-emotional outcomes when they are permitted to shift between their respective classical and quantum statistical continuum limits. Such lability, in contrast to the modeled network dynamics favored by Pessoa, enables scalable network partitioning and behavior reliant on both strong and weak nodal links and correlated activity observed and predicted for nervous systems.

Parameters included in Equations (1) through (10) satisfy the conditions for brain reorganization accompanied with learning. Each node may incorporate into networked patterns of different fitness or utility for cognitive-emotional representation and expression. Separate patterns and their partitioning may emerge for unique contexts that demand highly homogeneous to highly heterogeneous structure-function relationships. New nodes may be added to an evolving macro- or microcircuit, storing a set of nodes below the resource ceiling. Learning processes allocate a fitness parameter, *η*, for each node from a distribution *ρ*(*η*) holding values proportional or weighted to the net optimal or suboptimal gain of advantage perceived to be available from learning experiences. For some node *i*, *η_i_* is expressed as:

*η_i_* = |*p_i_P_i_*|. (11)

Argument *p_i_* in Equation (11) denotes the Bayesian probability that a node *i* is chosen and added to a network over other possible nodes in the larger neural field. A learner’s perceived or expected net payoffs, *P_i_*, relate experiential benefits and costs determined from arbitrary context-dependent probabilities [81,82,98,99], and bear a resemblance to, respectively, purely classical and quantum free-energy-based active inference models of decision-making and behavior [114,115].

Nodes compete and cooperate for cognitive-emotional circuit states of different fitness content, represented as network connections. Modal states are network solutions chosen more frequently by agents, and the nodes associated with those states, as well as others, accumulate new or newly recurrent connections, *m*, at each step *n* in the manner articulated by Equation (1). Recurrent connections in this definition differ from earlier treatments [81,82,87,98,99] to permit nodes to be revisited during cognitive-emotional processing without violating Equation (1). Positive or negative exponential changes in probability, Π*_i_*, for node *i* to accumulate new links indicates that use of the cognitive-emotional state corresponding to node *i* has become sensitized or habituated. Moreover, Cov[Π*_i_*,*Π_j_*] confers a practical determinant of connection strength between any two joined network nodes *i* and *j* and their respective cognitive-emotional state. Rising positive covariance between nodes implies escalating coupling strength resulting from learning mediated with Hebbian-like processes [8,13,81,82,83,84,98,99]. Rising negative covariance between nodes implies declining coupling strength that results from either extinction or inhibitory learning mediated with respective Hebbian- or nonHebbian-like processes. Former classes of adjustments in the strength of connections are analogous to active processes of memory suppression, instead of passive processes regulating normal or pathological memory decay. Furthermore, since an agent’s cognitive-emotional state might stay unchanged or might switch to another state of different fitness, state lability can be regarded as a transition between competing logic states or cognitive-emotional structures and functions, with Equation (12) agreeing with Landauer’s principle for memory storage devices [8,13,83,84,111,116,117],

*ε_i_* = −(1/*β*)ln2*η_i_*, (12)

where *β* = 1/*k*_B_*T, k*_B_ is Boltzmann’s constant and *T* is the absolute temperature of the local ambient environment. 

Because of the mathematical relationship between informational and physical degrees of freedom, energy level *ε_i_* in this context correlates with the minimum, although physically imprecise, energy absorbed and expended to “reset” or “erase” the state of node *i* [117]. Living or artificial agents may exist as endotherms, isotherms, and exotherms with their proximal environs, so modifications in energy level *ε_i_* mirror the composite system’s computational effort when shifting between nodes across an arbitrary time interval. This detectable thermal macroscopic energy matches the missing information or structural entropy tied to the precise internal microstate of node *i* and may be restated using Landauer’s principle without term *T* needed to manifest energy exchange [111,118]. Discrepancies in the value of *ε_i_* caused by distinctive informational and physical degrees of freedom may be corrected with limited satisfaction by universal and holographic entropy bounds. Bounds can be calculated, for instance, for a cell and its cellular compartments of definite physical dimensions and density [81,82,98,99], such as a neuron and its dendrites, soma, and axon. As an outcome of the preceding computational characteristics, network node *i* with lower *ε_i_* acting under recurrent autofeedback, such as an agent persistently selecting the same cognitive-emotional state, captures more links by maintaining higher Π*_i_*, higher positive Cov[Π*_i_*,Π*_i_*], and closer to perfect actual thermodynamic efficiency. This convention suits over-learned cognitive-emotional states computed by an agent that progressively burns less metabolic energy and tunes sparser structural resources than when computing under-learned cognitive-emotional states [81,82,98,99]. A nontrivial corollary then follows from this significant result. Network node *i* with higher *ε_i_* acting under recurrent autofeedback, such as an agent continuing to select the same cognitive-emotional state, begins to capture more links by also sustaining higher Π*_i_*, higher positive Cov[Π*_i_*,Π*_i_*], and improving actual thermodynamic efficiency. Such a scenario reflects the instance of cooperating weak nodal and microcircuit activity, which may prime and otherwise facilitate synaptic plasticity, contradicting Pessoa’s [1,29] belief that weak neuronal connections effect little-to-no influence over cognitive-emotional network structure and function.

### 5.2. State Transitions and Modularity of Hybrid Classical-Quantum Neural Fields

**Figure 1:**
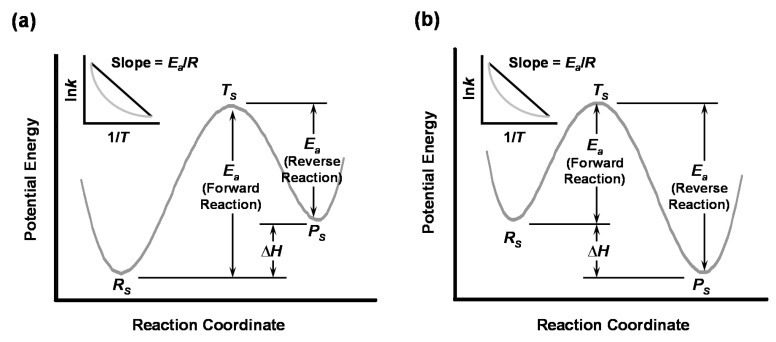
Model potential energy diagrams and Arrhenius plots for the rate or kinetics of cognitive-emotional state change. (**a**) Preferential attachment rules permit switching from one cognitive-emotional network node of higher fitness to another node of less fitness. Such computations may require “energy absorption” tantamount to endothermic chemical reactions. The initial fitter node, interpreted as a reactant (*R_s_*), maintains a lower potential energy. Forward transformation to a less fit node, interpreted as a product (*P_s_*), of higher potential energy may progress over or through the reaction barrier. A transition state (*T_s_*), also known as a reaction intermediate, mediates crossings over the reaction barrier. This scenario represents a classical reaction needing activation energy (*E_a_*) and a change in the system’s heat (∆*H*). Tunneling through the reaction barrier represents a quantum reaction. Arrhenius kinetics (inset) for classical reactions (black) form linear relationships between the kinetics of node switching (*k*) and the inverse of the system’s temperature (1/*T*). Quantum reactions (grey) render nonlinear Arrhenius kinetics between the same variables. (**b**) State transitions may also entail “energy dissipation” tantamount to exothermic chemical reactions. The initial less fit cognitive-emotional network node (*R_s_*) of higher potential energy transforms to a fitter node (*P_s_*) of lower potential energy with classical or quantum principles described for (**a**). Arrhenius kinetics (inset) of exothermic reactions return plots identical to those of endothermic reactions. Figure 1 and caption reproduced from [82] with permission.

Transitions between network nodes and microcircuits accompanying changes in cognitive-emotional states can be construed as endothermic or exothermic chemical reactions, as alluded to above. Switching from a state of higher fitness, and hence lower *ε_i_*, to one of lower fitness, and hence higher *ε_i_*, absorbs energy computationally equivalent to endothermic reactions (Figure 1) [82]; whereas, switching from a state of lower fitness, and hence higher *ε_i_*, to one of higher fitness, and hence lower *ε_i_*, dissipates energy computationally equivalent to exothermic reactions (Figure 1) [82]. Mathematical relations between state transitions and physicochemical processes, e.g., Refs. [81,82,98,99], affirm that thermodynamic independent quantum effects occurring with cognitive-emotional information processing will become exposed by nonlinear Arrhenius kinetics [82]. The Arrhenius equation ascertains the rate of a first-order reaction and assumes the formula:

(13) $$k=Ae^{-E_{a}/RT},$$

where *k* equals the rate constant, *A* equals a proportionality constant related to Boltz- mann’s and Planck’s constants, *E_a_* equals the activation energy, *R* equals the gas constant, and *T* equals the absolute temperature. When considering classical reactions, plotting ln*k* versus 1/*T* produces a linear function. Conversely, plots between the same two variables yield a nonlinear function for reactions involving quantum tunneling [82,119]. The biocomputational utility and relevance of the Arrhenius equation has attracted interest from several researchers who cleverly adapted the equation to examine and characterize the behavior and decision-making of thermally sensitive living organisms [82,120,121,122,123]. To execute such applications, the equation may be rewritten to take on the subsequent appearance:

*k* = (*k*_B_Γ/*h*)ƒ(*ε_i_*), (14)

where *k*_B_ equals Boltzmann’s constant, Γ equals the “critical tunneling field strength”, *h* equals Planck’s constant, and ƒ(*ε_i_*) equals the energy-dependent exponent determining network-node connectivity. The control or annealing parameter, Γ, substitutes absolute temperature *T* to be in alignment with published findings, e.g., Refs. [81,82,98,99], and is directly related to the critical condensation temperature *T*_C_ by Γ_C_ = (*T*_C_*k*_B_)/(*n*/ζ(3/2))^2/3^, where *k*_B_ is Boltzmann’s constant, *ζ* is the Riemann zeta function, and *n* is the “particle” density. Harnessing this interpretation of the Arrhenius equation dissociates classical from quantum computational phases in the operation and organization of complex networks, allowing for identification of cognitive-emotional processes based on free-energy values for work and state transitions. In such scenarios, activation energy *E_a_* signifies the intuition-deliberation activation energy, or threshold, analogous to that described by De Neys [124]. It is a set of discrete values *E_a_* = {*ε*${}_{a_{i}}$|*i* = 0, …, ∞} defined by differential energy or fitness levels *ε_i_* covering a set of values that partitions or gates network nodes *N* = {*n_i_*|*i* = 0, …, ∞} with the corresponding one-to-one mapped energy or fitness levels *Ε* = {*ε_i_*|*i* = 0, …, ∞}, as determined from Equation (1) through (10). The traditional Arrhenius equation, Equation (13), enables cognitive-emotional system switching, where changes in system heat *H*, temperature *T*, and energy *E* again obey Landauer’s principle and liberate free energy, *E*_free_ = *H* − *TS*, to perform classical work *W* through the work-energy theorem [84,125,126],

*W* = ∆*E* = ∆*E*_free_ = ∆*H* − *T*∆*S*. (15)

Equation (15) provides the foundation for a hybrid classical-quantum work-energy theorem,

*W* = ∆*E* = ∆*E*_free_ = ∆*H* − Γ∆*S*, (16)

also capable of enabling cognitive-emotional system switching by replacing *T* with the annealing parameter Γ. Recovery or maintenance of computational states, constituting classical to quantum Markov processes and a Szilárd engine-like model [84,126], occurs when lowering or raising entropy *S* drives respective “refrigeration” and “power” strokes from the input information (i.e., entropy defined in arbitrary information units) or energy reservoir to the output information (i.e., entropy defined in arbitrary information units) or energy reservoir, where entropy associated with prior stored information serves as a reference for error checking, and is then dissipated or extracted upon decision execution. Fidelity or trace distance between the two reservoirs, in accordance with the Second and Third Laws of Thermodynamics, connotes free energy for cognitive-emotional processing and the amount of entropy extracted and/or rejected to perform error-syndrome diagnosis and correction with power *P* = ∆*W*/∆*t*, (heat engine) efficiency *Φ* = *W*/*E*_input_, and (refrigeration engine or heat pump) coefficient of performance COP = *E*_output_/*W* [82,84,111,114,115]. The thermodynamic-sensitive classical limit reduces to classical power, efficiency, and performance functions; whereas, the thermodynamic-independent quantum limit relates work, power, and efficiency via the quantum work-energy theorem [127,128].

By re-envisioning Equation (7) with respect to Equations (13) to (16), the limits of work *W* may be scaled to *E_a_* of different computational regimes. The value of *E_a_* then decides the bounds for free energy needed to activate switches within and between regimes. In keeping with Equations (13) and (14), consider *E_a_* formulated as:

*E_a_* = nl(((*k*_B_Γ/*h*)ƒ(*ε_i_*))/*A*)^−*RT*^, (17)

and recall that when ƒ(*ε_i_*) = 1, a quantum Bose-Einstein computational phase dominates. Similarly, ƒ(*ε_i_*) << 1 and ƒ(*ε_i_*) >> 1 specify, respectively, classical Maxwell-Boltzmann and quantum Fermi-Dirac computational phases. The function ƒ(*ε_i_*) precisely and accurately determines *E_a_* thresholds for computational transitions and the ∆*E_a_* required to overcome gating mechanisms, leaving Equation (16) to now read:

*W* = ∆*E_a_* = ∆*E*_free_ = ∆*H* − Γ∆*S*. (18)

**Figure 2:**
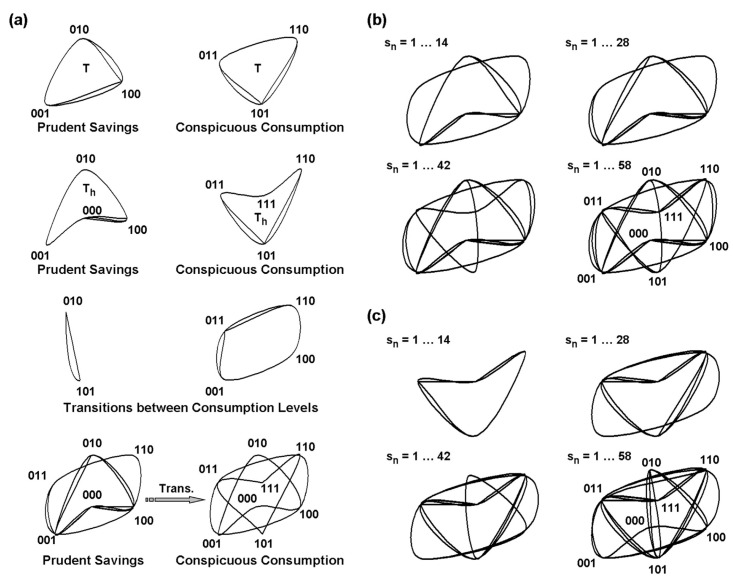
Some predictable topological features of evolving modular cognitive-emotional networks. (**a**) Nodes of 3D cognitive-emotional networks may self-organize into structure-function forms with several types of homology groups similar to simpler 2D graphical neural networks discussed by Pessoa. Nodes with weighted sum coordinate values of 1 or less represent prudent savings with lower fitness and bioenergetics resource availability. Those with weighted sum coordinate values of two or more represent conspicuous consumption with higher fitness and bioenergetics resource availability. Such diametric weighting profiles agree with neuroeconomic principles of cognitive computation [10,86]. Topologically invariant groups or manifolds of tetrahedral-like (T) (**top row**) and pyritohedral-like (T_h_) (**second row**) symmetry store different information of uniform categorical fitness for cognitive-emotional structure-function states in Riemannian space. Groups or manifolds storing information of mixed or fuzzy category fitness may transition between nodes signifying prudent savings and conspicuous consumption (**third row**). An incomplete network used by an agent evolving from embedded prudent-savings network groups or manifolds to conspicuous-consumption network groups or manifolds during learning experience (**bottom row**). (**b**,**c**) Examples of multistable architectures with embedded groups transforming over time, *s_n_*, into 3D computational networks with eight different nodes. The same agent built, searched, and employed both networks. Edges between computational network nodes in panels (**a**) through (**c**) denote an agent’s decision to switch between nodes. Recurrent cognitive-emotional processing overlap and might be obscured in network representations. Figure 2 and caption reproduced from [81] with permission.

**Figure 3:**
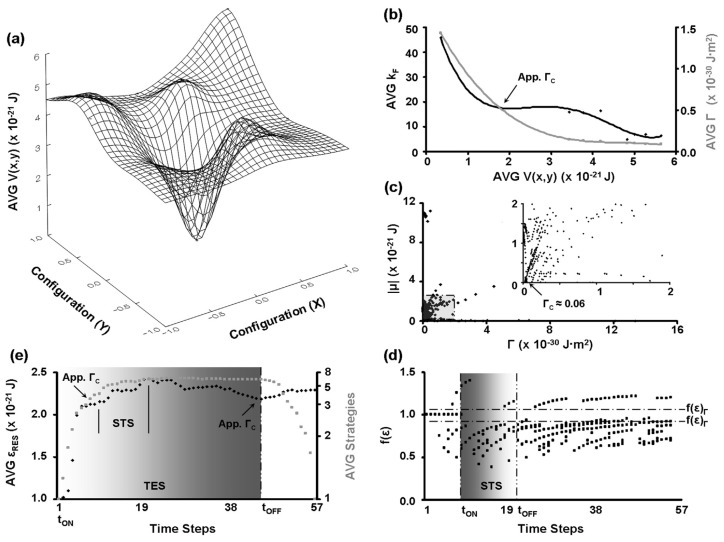
Predictable statistical mechanics properties of cognitive-emotional neural field partitioning and embeddedness. (**a**) Potential landscape with potential *V*(*x*,*y*) equaling energy level *ε* for each node in Riemannian space *R*. 3D structure of the network was transformed into an isomorphic *xy* (or *x*_1×2_) configuration for visualization of the landscape. Landscape attained deepest well at reconstructed coordinate (0, 0), which mapped onto modal node 000 with lowest *ε*. (**b**) Average final connectivity (*k*_F_) for evolving field nodes created a fourth-order polynomial distribution (black line). Most links amassed in the lowest average potential well of node 000. Bose-Einstein (BE) condensation for new connections emerged below the approximate “critical tunneling field strength”, App. Γ_C_. Average initial Γ (grey line) was proportional to *ε*, but diminished when energy levels gathered more links. Field nodes collecting about 20 connections changed from computational phases sensitive to thermodynamic limits into a BE condensate. (**c**) Precise plotting of |*μ*| against Γ established that Γ_C_ for node 000 was an order of magnitude lower than App. Γ_C_ in (**b**). Inset shows exploded view of area within grey-shaded box. (**d**) Rate, *f*(*ε*), at which the network modal node acquired or associated new connections showed the field began condensed on a single nodal solution located within the critical interval, *f*(*ε*)*_Γ_*. Learning or short-term sensitization (STS) reset BE computational phase to one dictated by Maxwell-Boltzmann statistics and other nodes with weights corresponding to conspicuous consumption fundamental groups. The abrupt conversion from quantum to classical computational models with different continuum limits is consistent with discontinuities in problem processing and restructuring of problem representation characteristic of intuitive problem-solving schemes. Learning offset accompanied return to *f*(*ε*) for BE statistics and preferred activity of node 000. Transitions from nodes of prudent savings to those of conspicuous consumption showed the field executed combinatorial and computational reorganization to effect context-dependent processing advantages. (**e**) Average residual energy, *ε*_RES_ (black dotted line), revealed annealing coincided with repeated associative trial-and-error nodal searches (TES) typical of deliberative problem solving. The field started at deepest average *ε*_RES_ common for condensation on a single node solution. STS reset AVG *ε*_RES_ to higher levels when average number of active nodes in the field grew (grey dotted line). TES annealed *ε*_RES_ to a minimum value near App. Γ_C_ with STS offset. Late onset BE condensation decreased average number of active network nodes until a prudent savings solution was chosen at quantum-search efficiencies. Returning to nodes of fundamental group prudent savings elevates labile context-dependent structure–function advantages. Figure 3 and caption reproduced from [81] with permission.

In this form, *W* is the amount of energy expended or absorbed by cognitive-emotional processes to resolve conflicts between competing structure-function outcomes. Cognitive-emotional networks are embedded as modular microcircuits (Figure 2 and Figure 3) in the global neural field of an agent effecting cognitive-emotional states and behavioral patterns. Work associated with ∆*E_a_* permits the system to switch between network nodes that define certain cognitive-emotional states. Larger amounts of available energy result in sudden, sizeable state transitions representative of discontinuous problem restructuring typically corresponding to cognitive-emotional intuition or insight [84]. Smaller amounts of available energy result in gradual, incremental decision transitions typically corresponding to cognitive-emotional deliberation or analysis [84]. While intuition processes may need a big initial investment and expenditure of energy to perform solution discovery, slower deliberation processes may demand greater total energy to reach solution discovery over more processing iterations. Levels of deliberative processing efficiency maintain predictable, direct relationships to time *T* and annealing parameter Γ, so that energy costs wasted on protracted, unsuccessful trail-and-error processing accumulate with each minor stepwise solution search, temporary processing stoppage, and solution pathway error correction. Risks inherent in this sort of processing mode only become offset when more rudimentary, automatic intuition attains an indefinite solution-discovery impasse for complex, hard cognitive-emotional problems [8,81,82,83,84,98,99,126].

In addition to energetics- or entropy-based network partitioning imposed by statistical mechanical or computational limits, topological and categorical traits may affect geometric network partitioning and embeddedness, and those traits may reciprocate to influence network function. Cognitive-emotional computational networks may be considered to be computational spaces, departing from Pessoa’s [1,29] simpler and perhaps conventional 2D graphical representations of cognitive-emotional networks. A finite homological group space, *S*_1_, is algebraically invariant from another finite homological group space, *S*_2_, of equal Euler characteristic,

*χ*(*S*) = Σ(−1)*^i^Β_i_*(*S*), (19)

where *Β_i_*(*S*) is the *i*th Betti or connectivity number of *S*. Here, *S* is any cognitive-emotional network in 3D Riemannian state space and *S*_1_ and *S*_2_ are compared state spaces. Organizing centers with two degrees of freedom for 3D state space dynamics may be assessed by Poincaré or fundamental groups, a set of loops in space *S* forming invariant topology. Different elementary groups within a state space indicate a manifold forms from nonsimply connected paths. Elementary groups of different homologies correspond to path connected or grouped strategies. Further, the trajectories connecting nodes represent the sequence of transitions or computations made during cognitive-emotional processing. Euler characteristics and internal angles for sets of 3D graph-like networks (Figure 2) indicate that hybrid quantum-classical networks may produce both unknotted and knotted geometric patterns as activity trajectories organize local and global networks over time [82,83,84,98,99]. The complexity of state-space structures proves sensitive to initial conditions without being fully reconstructed from those conditions. Qualities of this kind distinguish quasiperiodic Wolfram Class IV computations capable of emulations, such as universal Turing machines, cellular automata, and quantum machines [98,129,130]. Displays of periodic to quasiperiodic network activity correspond with respective Wolfram Class I, II, and IV computations. Such outcomes become crucial distinctions, as endpoint or thermostatic network topologies can yield identical Euler characteristics through different preceding cognitive-emotional processing dynamics. Thus, identifying periodic, quasiperiodic, and chaotic dynamics of network paths helps sort the types of cognitive-emotional computations executed by agents [129,130].

To determine 3D cognitive-emotional network dynamics, each subspace can be treated as a set of dissipative coupled *X*, *Y*, and *Z* (or corresponding arbitrary *x*_1_, *x*_2_, and *x*_3_) oscillators driven by finite cognitive-emotional processes. Both global (i.e., an entire network state space) and local (i.e., regions of a network state space) structure-function trajectories and their fundamental groups may be calculated from differential equations, or a procedure [131] justified by Takens’ embedding theorem [132] that relies on winding numbers and translation-invariant trajectory actions (i.e., winding number ratios). Frequencies may be established from discrete sections set at peak amplitudes of sinusoids for *X*, *Y*, and *Z* network dimensions, with winding numbers defined as,

*W* = *f_n,X_*:*f_n,Y_*:*f_n,Z_*, (20)

where *f_n_*_,*X*_, *f_n_*_,*Y*_, and *f_n_*_,*Z*_ are spline-smoothed sinusoids of *n*-fundamental frequency for each respective state space dimension. Trajectories show periodic behavior when *W* has rational values and show either quasiperiodic or chaotic behavior when *W* has irrational values [7]. Nonlinear coherence or coupling strength between oscillators,

*K* = |*C_XYZ_*|/|*A_X_*|*|*A_Y_*|*|*A_Z_*|, (21)

where *C_XYZ_* equals the cross-spectrum between state space dimensions and *A_X_*, *A_Y_*, and *A_Z_* equal autospectra for individual dimensions, differentiate quasiperiodic from chaotic activity [7]. For an absolute coupling strength interval of [0, 1] (i.e., maximum desynchronization at zero and maximum synchronization at one), chaos emerges for *K* > 0.25 [7]. Coherence values often fall well below that critical value, e.g., Ref. [98], giving networks with global irrational winding numbers an overall quasiperiodic computational structure with emergent strange nonchaotic attractors. By not developing chaotic structure-function patterns, agents maintain a remarkable capacity to avert maladaptive indeterminable (i.e., chaotic) Wolfram Class III computations that would force erratic cognitive-emotional network organization and behavior. Betti and primitive winding numbers, here winding numbers of fewer than three degrees of freedom, and their relationship to higher-order winding numbers, further show that certain families of network trajectory loops organize superposed dynamics of greater complexity. Fundamental groups, not considering torsion groups, of cognitive-emotional networks contain multiple elements and, therefore, signify non-simply connected spaces (i.e., manifolds) constructed from continuous closed paths. Closed paths represent the continual transition of one node to another and form geometries that reduce combinatorial and computational complexity by grouping and parsing nodes of similar categorical consumption demands, such as prudent savings or conspicuous consumption, together within computational space (Figure 2). Categories predictably aid in integrating relationships into canonical cognitive-emotional information, computations, and physical brain networks and their elements.

The hybrid classical-quantum cognitive-emotional networks discussed above map onto single-layer, flat Riemannian neural fields that may transform to deep and spherical neural fields, e.g., Ref. [133]. Mappings (Figure 3) illustrate distinct field characteristics which arise from the statistical nature and geodesics or trajectories of network structure-function evolution previously summarized for 3D graphical networks. Neural field dynamics may follow the given geodesic path:

*dσ*^2^ = ∑*g_ij_dx_i_dx_j_*, (22)

where scalar *dσ* equals the distance between two neighboring nodes *i* and *j,* with respective arbitrary energy or fitness levels *ε_i_* and *ε_j_* at respective arbitrary coordinates (*x*_1_, *x*_2_, *x*_3_) and (*x*_1_ + *dx*_1_, *x*_2_ + *dx*_2_, *x*_3_ + *dx*_3_) in Riemannian space *R* [134]. Here, *R* represents a potential surface, field, or landscape, where nodal energy or fitness levels create varying topological contours with local maxima and minima indicating the connectivity strength of a cognitive-emotional network obeying classical or quantum statistical behavior. Field contours, and thus network partitioning and embeddedness, are affected by the continuum limits of Bose-Einstein, Fermi-Dirac, and Maxwell-Boltzmann statistics, leading to adaptive or maladaptive structure-function reorganization of cognitive-emotional neural fields. Deep-welled fields nearing nodal condensations or singularities of highly compacted connections, such as those common to Bose-Einstein computations, may engage in fast, efficient, gradient-descent solution discovery for cognitive-emotional problems via diminished combinatorial and computational complexity. However, very strong coherence and periodicity can overly entrain macro- and microcircuits (e.g., fundamental groups or metrical neuromanifolds), locking local-to-global Wolfram class operations into inflexible attractor states and uncontrolled partition erosion into a large macrostate. If left unconstrained, such states may result in permanent system dysfunction or pathology uncharacteristic of healthy brains, adaptive natural or biological neural networks, and effective cognitive-emotional processing.

These aspects of brain structure and function, determined from neural field theory treatments, stress and complement other findings regarding the analytical insufficiencies and vulnerabilities common to graph-theory procedures, including the introduction of artifacts through invalid structure-function representational parcellation (or discretization) and activity correlation thresholds, often due to missing or improper physical constraints, dimensionality, and/or distinctions between scalar, vector, and tensor quantities [25]. Typical examples of graph-theoretic insufficiencies may be observed in resting-state fMRI and diffusion MRI signals fitted to classical entropic landscapes, similar to that shown in Figure 3, where regions of interest localized to brain default mode, frontoparietal, and cingulo-opercular networks exhibit connectivity and dysconnectivity associated with brain activity defined by maximum and pseudomaximum likelihoods or minimum probability flow methods [135]. Because hybrid classical-quantum local control parameters are sacrificed for higher-level deterministic spatiotemporal activity frequencies, statistical and energetic or entropic distributions of brain behavior other than Maxwell-Boltzmann ones become overlooked. Constructionist, generative field theories derived from more complex graphical representations thus better respect and rectify the physical nature and dimensionality of brains, retaining correct continuum limits and permitting diagrammatic analyses via nonstatistical and phenomenological eigenfunction-based coordinate and spectral domains unattainable with graph-theoretic measures. Such formulations may or may not resolve to, for example, neuronal, glial, or subcellular compartments, depending on the specified scale or gauge of parameter space and the method for normalizing (e.g., mean-field theory) over neural populations to obtain equations for local competitive-cooperative and weak-strong quantities, such as afferent activity, soma potentials, and firing rates [25]. For varying scale descriptions, activity across cortical convexities may be approximated with spatial structure eigenmodes governed by wave equations that notably solve the Helmholtz equation,

(23) $$\nabla^{2}u(r)=-k^{2}u(r)$$

where *r* designates spatial location, and spatial eigenmodes *u*(*r*) of brain activity are eigenfunctions of the Laplace-Beltrami operator $\nabla^{2}$ with eigenvalues *k*^2^, e.g., Refs. [136,137]. Low and high spatial frequencies, respectively, correspond to globally uniform and locally discrete structure-function features. Refinement of scale for higher spatiotemporal resolution, and ultimately cellular and subcellular representation, may be achieved with embedded state vectors that define local activity coupling and signal transmission along geodesic trajectories.

## 6. Summary

By ignoring neural field theories and physiologically derived constructs representative of neuronal plasticity, Pessoa, in his *The cognitive-emotional brain* [1], takes a dangerous, trending position in the neurosciences which disregards continuum effects on linear and nonlinear brain-network connectivity [133,138,139,140,141,142,143]. Although it is perhaps imperfect for some neurocognitive applications [144], absence of this content, so very important for understanding the dynamic structure-function embedding and partitioning of healthy and diseased brains, diminishes the rich competitive and cooperative nature of neural networks and consequently trivializes Pessoa’s arguments, and those made by like-minded contemporaries, e.g., Refs. [145,146,147,148], on the phylogenetic and operational significance of an optimally powerful integrated brain filled with both strong and weak neural connections. Riemannian neuromanifolds, containing limit-imposing (meta)plastic Hebbian- and antiHebbian-type control variables and other spatiotemporal neural parameters, simulate and model scalable network behavior difficult to capture from the simpler graph-theoretic analyses of imaged brain structure-function favored by Pessoa [1,28]. Field theories suggest partitioning and performance benefits of embedded cognitive-emotional networks optimally evolve between thermodynamics-sensitive exotic classical and quantum computational phases, where matrix singularities and condensations produce degenerate structure-function homogeneities dominated by strong connectivity unrealistic of healthy brains. When sustained for long periods of time in the quantum continuum limit of Bose-Einstein statistics, such distributed, over-entrained macrostate patterns of cognitive-emotional brain dynamics become inextricably driven by excessive, strong local neural connections and correlated activity, juxtaposing with Pessoa’s belief that embeddedness tends to maximize brain organization and operation. Instead, multistable network partitioning across and within classical and quantum statistical regimes, in comparison to unconstrained embeddedness, is required for effective cognitive-emotional network structure and function, and this phenomenon requires greater attention in our new era of neuroscience.

One topic of particular interest for current and future neuroscience is connectome research, such as is being produced at the NIH Human Brain Project and the Blue Brain Project, as well as other related high-throughput computational work [149,150,151]. Growing ambitions and the proliferation of brain connectome research has inspired, over decades, expanded use of simpler, albeit scalable, graph-theory approaches for the more-or-less tractable structure-function mapping and analyses of micro-, meso-, and macroscale nervous system connectivity. The computing- and data-intensive nature of connectome studies, as well as their positive contributions to the understanding and treatment of human disease and injury [145,149,152], to some extent justify, and even dictate, the development and application of 2D or 3D graphs to characterize multilevel, distributed brain architectures, pathways, topographies and topologies, and functions. However, as discussed throughout this paper, these paradigm conventions come with substantial deficits in theory- and experiment-backed details revealed with clinical and laboratory techniques including imaging, electrophysiological, histological, bioinformatics, and other methods. These deficits include the failure to adopt spatiotemporal (meta)plastic control parameters and multi-omics data elements (e.g., connectomics, genomics, epigenomics, transcriptomics, proteomics, metabolomics, exposomics, etc.) necessary for accurate, precise, and meaningful creation and interpretation of brain models and simulations, e.g., Refs. [25,153,154].

Accordingly, ongoing efforts seek to either adapt graph-theoretical constructs or to replace them with more sound neural fields which better incorporate realistic biophysical properties and mechanisms, physical structure, and spatial geometry into brain connectomics parameters [152,155,156,157,158,159,160]. Newer connectomic parameters now employed to enhance graph-theoretic brain structure-function models include wave-particle-defined neural conduction and entropy-quantified activity patterns. These sorts of parameters help normalize the brain connectome across network scales, while also proffering physiologically relevant explanations for neurocognitive processing speeds and the emergence of frequency-specific network cores, such as localized normal and pathological brain rhythms and coupled oscillations. State-of-the-art neural field frameworks nevertheless often exceed the cruder capabilities, descriptive validity, and power of graphical brain representations. For instance, trends in spectral factorization of brain fields enable unrivaled systematic representation and analyses of local-to-global neural connectivity and dynamics. Advantages include the promotion of testable predictive and attributional inferences about connectome eigenmodes and activity indices, such as electromagnetic spectra, evoked responses, activity coherence and propagation, self-connectedness, and causal mechanisms significant for brain function and (re)wiring, as well as the diagnoses, monitoring, and treatment of brain diseases and injury. Lastly, the use of eigenmodes to portray brain structure and function grants a precise, accurate, and flexible means of mapping resting and stimulus-induced spatiotemporal dynamics within the continuum limits of fine- to course-scale fields, yielding discrete to dispersed canonical or summed patterns of neural organization and operation. Such mathematical depictions of brain connectomes, as before illustrated with the hybrid classical-quantum neural field model, show that complex brain structure-function relationships may be neither reduced to one simple fundamental mapping nor expanded to infinite fundamental mappings. Rather, the healthy brain connectome and its behavior reside in a delicate structure-function balance, directed and bounded by continuum limits set by physiological constraints usually unappreciated by ordinary graph theories (Box 1).

Box 1 **Summary Highlights.**

What are the main findings?

- Neuromanifolds with metaplastic control variables simulate scalable network behavior.
- Simulations identify structure-function limits unresolved by graph analysis of imaged brains.

What is the implication of the main finding?

- Embedded brain networks optimally evolve between exotic computational phases.
- Exotic phases yield degenerate structure-function homogeneities unrealistic of healthy brains.
- Some network partitioning, not unconstrained embeddedness, maximizes brain functionality.

## Data Availability

Not applicable.

## Glossary

- *Classical and Quantum Computational Phases* are computational states of complex systems, such as natural or technological neural networks, which obey respective classical or quantum statistical mechanics.
- *Classical Networks* are complex biological and technological networks whose connectivity tends to follow Maxwell-Boltzmann classical statistics rather than Bose-Einstein or Fermi-Dirac quantum statistics.
- *Combinatorial and Computational Complexity* are classes of complexity defined or measured by a system’s structural (e.g., degree of embeddedness or partitioning) and processing features (e.g., resource allocation and capacity, processing speed, etc.).
- *Degenerate Homogeneity* is a limiting condition of network structure-function which converges onto a single state, value, or distribution.
- *Deterministic Singularity* is the operational and/or organizational convergence of a complex biological or technological classical network onto one indistinguishable macroscopic state. The connectivity of these classical networks obeys Maxwell-Boltzmann, Shannon-Einstein, and other classical statistics when nodal strength is described as separate fitness or energy levels and nodal links take on the identity of particle states functioning under associative-like preferential attachment rules. In such cases, a thermodynamic control parameter dictates system behavior and is often represented with a computational annealing parameter, such as space or time. Singularity occurs in the thermodynamic limit.
- *Effectively Connected Network* is a brain network showing correlated activity supported by known anatomical connectivity.
- *Eigenfunctions* are a type of eigenvector with nonzero functions acted on by linear functions (e.g., matric operators) in a defined function space which render scalar eigenvalues designating amplitudes for eigenfunctions.
- *Equipotentiality* is neurophysiological and behavioral phenomena, often attributed to Karl Spencer Lashley, which enable intact cortical brain areas to perform functions, such as memory, lost by other cortical brain areas due to physical damage. The concept of equipotentiality supports and is consistent with overlapping or embedded distributed parallel networks capable of plastic reorganization and functional redundancy. Originally, equipotentiality and the Law of Mass Action were presented as counterarguments to observed structure-function relationships strictly localized to discrete brain areas, such as speech production and comprehension associated with brain language centers.
- *Euler Characteristic or Number*, named after Leonard Euler, is an algebraic topological measure of spatially invariant structures with a various properties, including, but not limited to, properties for summation, products, fibration, and covering.
- *Fitness Level* is the nodal weighting parameter for networks governed by associative-like preferential attachment rules.
- *Geodesics* are paths in Riemannian space that connote spatiotemporal changes in field properties, such as energy or entropy levels which shape field contours and other topological features.
- *Graph Theory* is the mathematical application of graphs and their properties to describe the behavior and organization of computational objects, such as natural and artificial neural networks. These objects comprise a collection of nodes or vertices connected by directed or undirected edges.
- *Hebbian Learning Rules*, named after Donald Hebb, are iterative adaptive control mechanisms utilizing activity-dependent bidirectional or dual-processes associative learning rules to either strengthen or weaken nodal connections of an associative (biological or technological) network. The set of learning rules (e.g., cooperativity, coactivity or associativity, synaptic or nodal efficacy/weight, etc.) governing this kind of feedback/feedforward regulation, whether at neuronal synaptic junctions or other nodal forms of computational circuitry, may fall under different subclassifications, such as nonlinear and nonstochastic Hebbian learning, dynamic stochastic synaptic plasticity, spike timing-dependent plasticity, and quantum bidirectional plasticity. When natural or simulated synaptic plasticity occurs that violates Hebbian rules, it is said to be antiHebbian in nature.
- *Metaplasticity* is the modulation of synaptic plasticity caused by neuromodulatory chemicals and cellular processes.
- *Model-Free Functional Network* is a biological or technological network organized by activity correlations without presumptive physical connectivity.
- *Multistability* refers to the activity of a dynamic system with topological minima and maxima representative of different, adaptive system combinatorial and computational states. Systems that are multistable maintain a capacity to organize and operate in states of differential fitness to try to maximize system outcomes under varying contexts.
- *Neural Field Theory* represents a broad class of mathematical or computational models of brain network organization and operation. This class of models, including mean field theory, relativistic Shannon-Einstein theory, quantum field theory, and other models, employs physiologically relevant control variables, such as synaptic plasticity rules, to approximate the full dynamic range of neural structure-function evolution across different spatiotemporal (neuromanifold) scales. Besides successfully modeling biologically observed local and global brain behavior via relativistic and quantum physics, field theories remain unmatched in their capacity to predict the outcomes of control variable perturbation on brain state.
- *Neuroeconomic Principles of Cognitive Computation* enlist cellular energetics and organismal bioenergetics concepts and metrics to determine energy or entropy savings and consumption associated with the respective thermodynamic or informational work of scalable cell-based cognitive processes.
- *Pleuripotency*, sometimes reported as being first described by Rafael Lorente de Nó, is neurophysiological phenomena similar to equipotentiality that endow brain cells, particularly neurons, and circuits, such as cortical columns, with the ability to perform in highly plastic multifunctional capacities which assume traits found for various other cell types and different local brain circuitry. Pleuripotency depends on the nature of structure-function connectivity and may adapt to both weakly and strongly correlated neuronal activity.
- *Poincaré or Fundamental Groups*, named after Henri Poincaré, are topologically algebriac classes of homotopic space, such as Minkowski spacetime isometries, that define, for example, simply connected (or continuous path-connected), nonsimply connected, and disconnected Riemannian space through invariant or equivalent loop structures.
- *Probabilistic Condensation* is the operational and/or organizational convergence of a complex biological or technological quantum network onto one indistinguishable macroscopic state. The connectivity of these quantum networks obeys Bose-Einstein statistics when nodal strength is described as separate fitness or energy levels and nodal links take on the identity of particle states functioning under associative-like preferential attachment rules. In such cases, a thermodynamic control parameter dictates system behavior and is often represented with a computational annealing parameter, such as space, time, or the “critical tunneling field strength”. Condensation occurs in the thermodynamic limit.
- *Quantum Networks* are complex biological and technological networks whose connectivity tends to follow either Bose-Einstein or Fermi-Dirac quantum statistics rather than Maxwell-Boltzmann classical statistics.
- *Quantum or Bloch Spheres*, named after Felix Bloch, are quantum-mechanical geometric visualizations mapped onto a unitary 2-sphere Riemannian space with mutual orthogonal basis state vectors serving as antipodes (or poles). Spheres correspond to 2D Hilbert spaces, with surface points being pure quantum states and interior points being mixed ones.
- *Resting State or Intrinsically Connected Neural Activity* is correlated spontaneous wakeful brain activity.
- *Riemannian Neuromanifold*, named after Bernhard Riemann, is a neural field or matrix that conforms to Riemannian geometry of curved space, such as positive and negative curvature, not explained by flat 3D Euclidean geometry. The structure-function relationships of such manifolds are simplified and more fully captured by expanding the Pythagorean theorem to arbitrary dimensions with a metric tensor that describes relative local and global properties of a field.
- *Small-World and Scale-Free Networks* are classes of complex biological and technological networks with unique structure-function properties, such as the distribution, number, and local size of nodal neighborhoods, the length of connecting paths, and network growth rates. The global behavior and organization of a complex network tends to evolve according to a logarithmic law of connectivity for small-world objects and to a power law of connectivity for scale-free objects.
- *Structure–Function Embedding and Partitioning* is the respective encapsulation, comparmentalization, discetization, or parcellation of a biological or technological network into structural and functional units.
- *Thermodynamic Bound* is the structure-function limit forced by a control parameter on a biological or technological system, such as natural and artificial neural networks. Control parameters may be actual absolute ambient temperature or computational annealing parameters, such as space, time, or the “critical tunneling field strength”.
- *Topographic Network Theory* is the physical or logical description of network structure and function using various mathematical models, including, among other frameworks, mean-field, Shannon-Einstein, and quantum theories.
- *Wolfram Complexity Classes*, named after Stephen Wolfram, are a set of four computational complexity classes numbered in ascending order of complexity. Class I complexity displays simple behavior, with nearly all initial conditions driving the system toward the same uniform final state. Class II complexity returns many different final states, with each containing a certain set of simple structures that either always remain the same or cycle through structural iterations after repeated computational steps. Class III complexity shows more complicated behavior, appearing almost random with some simple small-scale structural elements always present. Class IV complexity presents a mixture of ordered and random behavior, with local simple structures which interact in a highly complex manner.
